# Unilateral Diffuse Alveolar Hemorrhage in a Polysubstance User: A Case Report

**DOI:** 10.7759/cureus.100841

**Published:** 2026-01-05

**Authors:** Joana Lopo, Ana Margarida Morgado, Paula Nogueira, Pedro Reboredo, Fatima Cereja

**Affiliations:** 1 Internal Medicine, Unidade Local de Saúde do Algarve, Faro, PRT

**Keywords:** cocaine-related disorders, diffuse alveolar hemorrhage, flexible bronchoscopy, ilicit drugs, severe respiratory distress syndrome

## Abstract

Diffuse alveolar hemorrhage (DAH) is a rare but severe condition characterized by bleeding into the alveolar spaces, which can lead to acute respiratory failure. The most common causes are vasculitides and autoimmune diseases, but the growing presence of illicit drugs such as cocaine and opioids in the lives of young adults has turned these into risk factors as well. We present a 32-year-old man with a history of heavy daily alcohol consumption (>350 g/day) and inhalational use of cannabis, cocaine, and heroin. He was found unconscious at home, with partial recovery of consciousness after administration of naloxone, leading to the assumption of an opioid overdose. He subsequently developed hypoventilation of the left hemithorax, severe global respiratory failure, and required invasive mechanical ventilation. Chest computed tomography revealed confluent consolidations involving almost the entire left lung, consistent with diffuse alveolar hemorrhage. The diagnosis was confirmed by bronchoscopy with hemorrhagic bronchoalveolar lavage. Treatment included ventilatory support, empiric antibiotics, and supportive therapy for withdrawal. After a favorable clinical course, he was discharged with follow-up at a specialized support center. Drug-induced DAH represents a diagnostic and therapeutic emergency. Polysubstance use, particularly the combination of cocaine and opioids, can cause alveolar injury and diffuse hemorrhage. Early recognition and intensive supportive care are essential for recovery. This case highlights the importance of considering DAH in patients with drug intoxication, especially in the context of simultaneous substance use. A high level of clinical suspicion is required for prompt diagnosis and appropriate intervention.

## Introduction

Diffuse alveolar hemorrhage (DAH) is a potentially fatal clinical-radiological syndrome characterized by blood extravasation into the alveolar space, resulting in hypoxemia, diffuse radiological infiltrates, and occasionally hemoptysis. Typically presents with bilateral involvement, reflecting its diffuse involvement of the pulmonary microvasculature; however, unilateral presentations, although uncommon, have been described. These atypical cases pose a diagnostic challenge and are frequently misinterpreted as pneumonia, pulmonary edema, or focal hemorrhage, leading to inappropriate or delayed management. DAH represents a constellation of manifestations that share a common pathophysiological mechanism, injury to the alveolar-capillary barrier, but may have distinct etiologies, including antineutrophil cytoplasmic antibodies (ANCA)-associated vasculitis, anti-glomerular basement membrane syndrome, connective tissue diseases, infections, coagulopathies, and toxic exposures such as illicit drugs and medications. Substances such as cocaine (particularly in its crack form) can induce DAH through direct injury to the alveolar-capillary membrane [1].

Although classic cases involve vasculitides, there is a growing number of reports describing alveolar hemorrhage associated with illicit drug use. Recent literature documents episodes of DAH triggered by cocaine, opioids (such as heroin or fentanyl), and even cannabis [2]. Unilateral DAH in the setting of illicit drug use may result from preferential aspiration, asymmetric exposure to inhaled toxins, focal vascular injury, or regional ventilation-perfusion mismatch [3-5].

Despite being widely recognized by intensive care and pulmonary physicians, the true incidence of DAH remains difficult to determine due to underreporting, etiological heterogeneity, and variable clinical presentation. Observational studies estimate that DAH is a rare cause of acute respiratory failure requiring admission to intensive care units, although this proportion may be higher when toxic and iatrogenic causes are included [6]. The majority of the cases described are related to autoimmune diseases, while infectious and drug-induced causes represent increasing proportions, particularly among young adults [2,7].

Mortality associated with DAH varies widely, ranging from 20% to 50%, depending on the etiology, time to diagnosis, and severity of respiratory failure [8]. Of particular relevance in recent years is the rising number of cases associated with illicit substance use, notably cocaine, heroin, synthetic cannabinoids, and potent opioids. Inhalational drug use can trigger acute injury to the alveolar-capillary membrane, an intense inflammatory response, and a sudden increase in vascular permeability, culminating in alveolar hemorrhage. This mechanism has been documented especially in polysubstance users, a group whose prevalence has been increasing globally [9].

Nonspecific clinical presentation, which may include sudden dyspnea, acute anemia, diffuse alveolar infiltrates, and, in some cases, hemoptysis, requires a high index of suspicion for early diagnosis. Chest computed tomography (CT), often demonstrating ground-glass opacities and diffuse consolidations, and bronchoscopy with bronchoalveolar lavage play a central role in diagnostic confirmation. Given the high mortality and rapid clinical progression, prompt recognition of DAH and early initiation of treatment directed at the underlying cause are essential to improve prognosis.

In this context, illicit drug-induced DAH constitutes an emerging challenge in clinical practice, particularly among young adults without a history of autoimmune disease. Cases such as the one presented underscore the need to raise clinician awareness of this etiology, enabling timely diagnosis and appropriate intervention.

## Case presentation

A 32-year-old man with no known medical history but with a history of heavy daily alcohol consumption (>350 g/day) and inhalational use of cannabis, cocaine, and heroin, was found unconscious at his residence by family members. At the scene, due to non-response, naloxone was administered by the emergency team, with partial recovery of consciousness leading to the assumption of opioid overdose at that time.

On physical examination, there was a marked reduction in vesicular murmur throughout the left hemithorax, along with low peripheral oxygen saturation. Arterial blood gas (ABG) analysis revealed severe respiratory failure (ABG: pH 7.12, partial pressure of carbon dioxide (pCO₂) 62 mmHg, partial pressure of oxygen (pO₂) 51 mmHg, saturation 81%). Laboratory studies were notable for hemoglobin drop (140 to 127 g/L) and leukocytosis, increasing from 14,400 to 19,000 cells/µL. Because the C-reactive protein (CRP) level rose from 124 to 338 mg/L, empiric antibiotic therapy with ceftriaxone was initiated due to possible aspiration. Creatine kinase (CK) elevation was related to prolonged immobilization as well as to illicit substances that cause intense vasoconstriction, increased muscle activity, and hyperthermia, leading to muscle injury (Table 1). Due to rapid respiratory deterioration, invasive mechanical ventilation was required, and orotracheal intubation was performed.

**Table 1:** Blood analysis at admission (day 0) and 24 hours later (day 1)

Parameters	Patient Values (Day 0)	Patient Values (Day 1)	Reference Ranges
Hemoglobine	140 g/L	127 g/L	130 - 170 g/L
Leucocytes	14.4 x10^9^/L	19.4 x10^9/L	4.0 - 10.0 x10^9^/L
Platlets	313 x10^9^/L	271 x10^9/L	150 - 400 x10^9^/L
Creatine kinase	2243 UI/L		30 - 200 UI/L
C-reactive protein	124 mg/L	338 mg/L	< 5 mg/L

**Figure 1:**
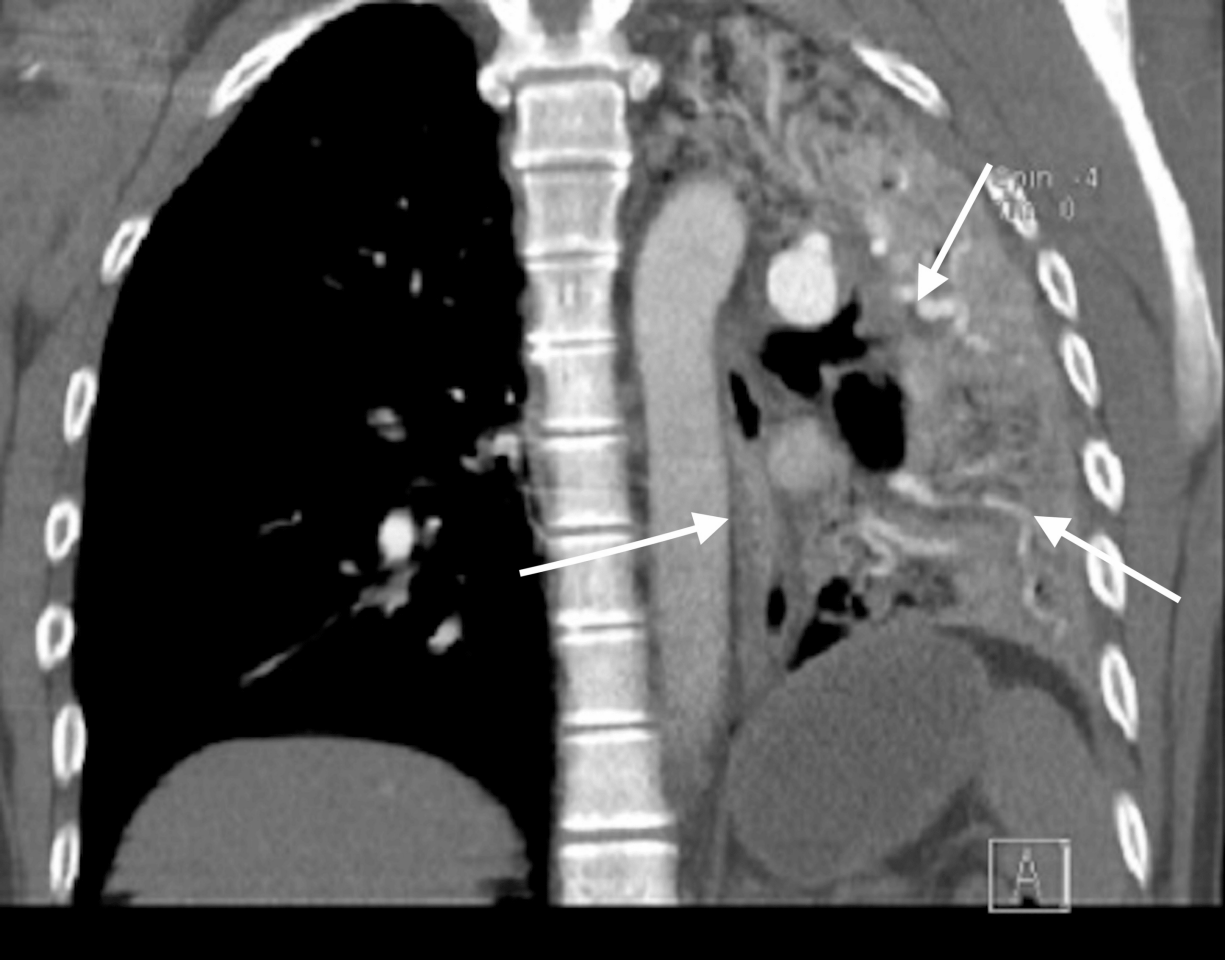
Chest CT (coronal view) showing reduced volume of the left lung with near-total involvement by confluent consolidations extending to the pulmonary periphery, consistent with diffuse alveolar hemorrhage. No intraluminal filling defects are observed that would suggest pulmonary embolism (PE). There is a decrease in volume of the left lung with almost total involvement by confluent consolidations extending to the pulmonary periphery, compatible, within an appropriate clinical context, with alveolar hemorrhage associated with drug use. The tracheobronchial tree is patent up to its tracheal bifurcation. No supraclavicular, axillary, hilar, or mediastinal lymphadenopathy of significance by size criteria is observed. The heart and great vessels show no abnormalities. There is no pericardial effusion.

**Figure 2:**
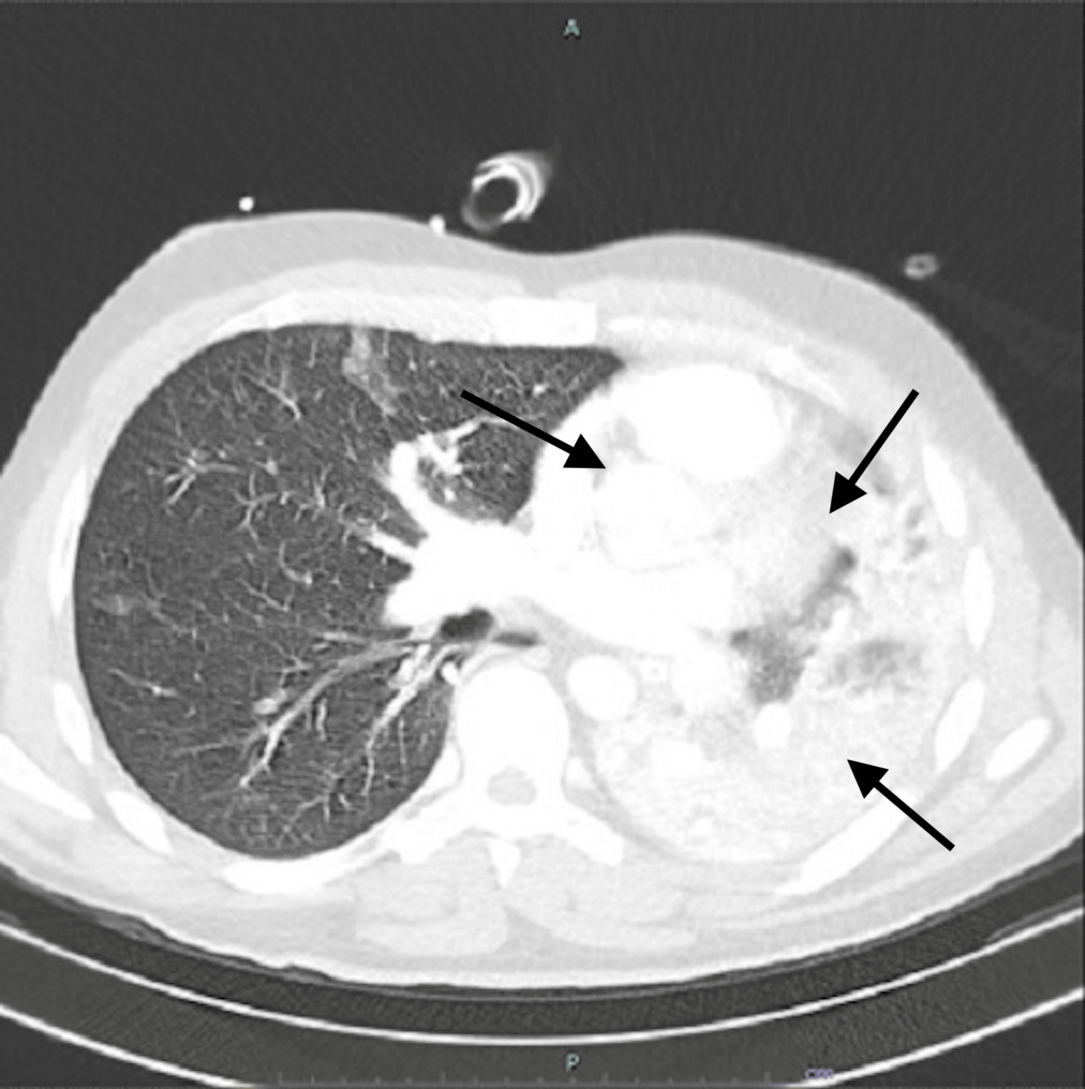
Chest CT (axial view) showing reduced volume of the left lung with near-total involvement by confluent consolidations extending to the pulmonary periphery, consistent with diffuse alveolar hemorrhage.

CT revealed reduced volume of the left lung with near-total involvement by confluent consolidations extending to the pulmonary periphery, findings that, in the appropriate clinical context, were consistent with diffuse alveolar hemorrhage associated with drug use (Figures 1, 2). The unilateral lung involvement in polysubstance abusers can be explained on the basis of aspiration of vomitus on one side of the lung if the patient is lying with one side down; however, in such a scenario, the aspirated material may be visualized in the airways, which was not seen in the present case. Unilateral acute respiratory distress syndrome (ARDS) as a result of unilateral pulmonary embolism has been kept as a possibility in the present case.

Among the complementary diagnostic tests performed, bronchoscopy was particularly noteworthy, revealing bloody return on bronchoalveolar lavage. The bronchoalveolar lavage showed increased red blood cells (48 cells/mm^3^), and with the reduction of serum hemoglobin, the diagnosis of DAH was assumed.

In the serological/immunological workup, it was possible to diagnose an acute infection of syphilis, as shown in Table 2. All the other possible causes came out negative. However, as the patient was already receiving ceftriaxone for suspected aspiration (given the unknown time interval until he was found and the presence of elevated inflammatory markers), it was decided not to modify the antibiotic regimen.

**Table 2:** Serological and immunological study *Electrochemiluminescence method; **Immunochromatography method HBsAg: hepatitis B surface antigen; HBsAb: hepatitis B surface antibody; HBcAb: hepatitis B core antibody; HCV, ab: hepatitis C virus antibody; ag: antigen; VCA: viral capsid antigen; EBNA: Epstein-Barr virus nuclear antigen; ANA: antinuclear antibody; ANCA: antineutrophil cytoplasmic antibodies; MPO: myeloperoxidase; PR3: proteinase 3

Parameters	Patient Values	Reference Ranges
HIV 1+2, ab/ag*	Negative	-
Treponema pallidum, IgG+IgM*	Positive	-
Rapid plasma reagin	Positive, 1/8	-
HBsAg*	Negative	-
HBsAb*	Negative	-
HBcAb*	Negative	-
HCV, ab*	Negative	-
*Legionella pneumophila*, ag**	Negative	-
*Streptococcus pneumoniae*, ag**	Negative	-
Epstein-Barr VCA, IgG**	Negative	< 0.21
EBNA, IgG**	Negative	< 0.21
Epstein-Barr VCA, IgM**	Negative	< 0.19
*Cytomegalovirus*, IgM	Negative	<6 UA/ml
*Cytomegalovirus*, IgG	Negative	< 0,90
ANA	Negative	-
MPO-ANCA	Negative	< 10
PR3-ANCA	Negative	< 5

After five days in the intensive care unit, the patient showed progressive improvement in respiratory function and laboratory parameters. He was extubated when clinically appropriate and, after stabilization, he was discharged from the hospital, 15 days after admission, with a referral and follow-up at a specialized addiction support center.

## Discussion

Drug-induced DAH is a rare but potentially fatal entity. Cocaine, particularly in inhaled forms (crack or powder), is among the most frequently reported substances [6]. Pulmonary injury results from multiple mechanisms, often acting together, including drug-induced vasoconstriction, direct damage to the alveolar-capillary endothelium, and increased capillary permeability, leading to blood extravasation into the alveoli. In the case of opioids, there are reports of alveolar hemorrhage following heroin and fentanyl overdose, possibly related to respiratory depression, hypoxia, and increased negative intrathoracic pressure during apnea, as well as secondary alveolar injury [2].

Furthermore, as previously described, recent reports suggest that the combination of substances (polysubstance use) may have a synergistic effect in inducing DAH [4]. In our patient, the reported use of cocaine and heroin may have contributed additively or synergistically to the hemorrhagic event.

Although less common, cannabis has also been associated with DAH. Lung injury may be related to the toxic effects of smoke (e.g., contamination, combustion products) on the alveolar membrane [10]. In the present case, cannabis use may have been a contributory or predisposing factor, although the exact mechanisms remain poorly defined.

In unilateral DAH related to illicit drug use, the fundamental problem is toxic injury to the alveolocapillary membrane, leading to structural failure of the barrier that normally prevents blood from entering the alveoli. The mechanism is not unique to the unilateral form; rather, unilateral involvement reflects regional vulnerability to the same injurious processes. When drugs are inhaled or injected, they reach the pulmonary microcirculation at high concentrations. Many of these substances and their adulterants cause direct endothelial toxicity, leading to apoptosis or necrosis of capillary endothelial cells. This disrupts tight junctions and weakens the capillary wall. Once this structural damage occurs, the alveolocapillary barrier loses its integrity, and red blood cells leak freely into the alveoli [3,11].

Cocaine and similar sympathomimetic drugs also cause intense pulmonary vasoconstriction and sudden elevations in pulmonary capillary pressure. This creates capillary stress failure, in which mechanical forces stretch the already injured capillary walls. Microscopic breaks develop in the endothelium and basement membrane, allowing intact red blood cells to pass into the alveolar spaces. This mechanism explains why hemorrhage can occur abruptly, even without inflammation. The unilateral distribution of hemorrhage is thought to result from uneven drug deposition, asymmetric pulmonary blood flow, regional differences in vascular tone, or pre-existing lung injury on one side [11]. 

The diagnosis of DAH requires a high degree of clinical suspicion, particularly in patients with a history of illicit substance use. Chest CT is an essential diagnostic tool, typically showing ground-glass opacities, alveolar consolidations, interlobular septal thickening, and other compatible findings [12]. Bronchoscopy with bronchoalveolar lavage is considered the gold standard for confirmation, demonstrating progressively more hemorrhagic return and, ideally, macrophages with hemosiderin deposition [13]. In addition, it is important to exclude other causes, such as vasculitides, anti-glomerular basement membrane syndrome, infections, and heart failure.

The therapeutic approach to drug-induced DAH combines intensive supportive care (mechanical ventilation, oxygenation), treatment of the underlying cause (cessation of the offending drugs), and, frequently, corticosteroids. The use of steroids is not universally standardized, but in many cases, high doses are administered to control inflammation and limit alveolar damage. In reported cases, withdrawal of the causative substance and respiratory support have resulted in complete or partial recovery [2].

In our case, the combination of ventilatory support, empiric antibiotic therapy, and intervention for substance dependence was successful, leading to recovery and hospital discharge. The importance of a structured follow-up plan to prevent relapse and recurrent hemorrhagic episodes is emphasized.

## Conclusions

This clinical case is relevant as it highlights the need for a high index of suspicion for DAH in patients with overdose or illicit drug use, particularly in situations of unexpected respiratory deterioration. It underscores the importance of recognizing that polysubstance use (alcohol, cannabis, cocaine, heroin) can mask or complicate the diagnosis, since symptoms may overlap with other types of pulmonary injury or toxidromes, and that early intervention (bronchoscopy and intensive supportive care) can be decisive for survival and recovery.

Integration among intensive care, pulmonology, toxicology, and addiction services is vital for comprehensive patient management. Nevertheless, because is not common , clinicians should maintain a high index of suspicion for DAH in patients with drug use and respiratory deterioration, even in the absence of obvious autoimmune causes.
